# The Interpersonal Neurobiology of Intersubjectivity

**DOI:** 10.3389/fpsyg.2021.648616

**Published:** 2021-04-20

**Authors:** Allan N. Schore

**Affiliations:** UCLA David Geffen School of Medicine, Los Angeles, CA, United States

**Keywords:** intersubjectivity, right brain, right temporoparietal junction, nonverbal communication, interpersonal synchrony, interbrain synchronization, brain laterality development, psychotherapy

## Abstract

In 1975, Colwyn Trevarthen first presented his groundbreaking explorations into the early origins of human intersubjectivity. His influential model dictates that, during intimate and playful spontaneous face-to-face protoconversations, the emotions of both the 2–3-month-old infant and mother are nonverbally communicated, perceived, mutually regulated, and intersubjectively shared. This primordial basic interpersonal interaction is expressed in synchronized rhythmic-turn-taking transactions that promote the intercoordination and awareness of positive brain states in both. In this work, I offer an interpersonal neurobiological model of Trevarthen’s intersubjective protoconversations as rapid, reciprocal, bidirectional visual-facial, auditory-prosodic, and tactile-gestural right brain-to-right brain implicit nonverbal communications between the psychobiologically attuned mother and the developing infant. These co-constructed positive emotional interactions facilitate the experience-dependent maturation of the infant’s right brain, which is in an early critical period of growth. I then address the central role of interpersonal synchrony in intersubjectivity, expressed in a mutual alignment or coupling between the minds and bodies of the mother and infant in face-to-face protoconversations, as well as how these right brain-to-right brain emotional transmissions generate bioenergetic positively charged interbrain synchrony within the dyad. Following this, I offer recent brain laterality research on the essential functions of the right temporoparietal junction, a central node of the social brain, in face-to-face nonverbal communications. In the next section, I describe the ongoing development of the protoconversation over the 1st year and beyond, and the co-creation of a fundamental energy-dependent, growth-promoting social emotional matrix that facilitates the emergence of the highly adaptive human functions of mutual play and mutual love. In the final section, I discuss the clinical applications of this interpersonal neurobiological model of intersubjectivity, which has a long history in the psychotherapy literature. Toward that end, I offer very recent paradigm-shifting hyperscanning research that simultaneously measures both the patient and therapist during a psychotherapeutic interaction. Using the Trevarthen’s two-person intersubjective model, this research demonstrates changes in both brains of the therapeutic dyad and the critical role of nonverbal communications in an emotionally-focused psychotherapy session. These studies specifically document interbrain synchronization between the right temporoparietal junction of the patient and the right temporoparietal junction of the clinician, a right brain-to-right brain nonverbal communication system in the co-constructed therapeutic alliance. Lastly, I discuss the relationship between the affect communicating functions of the intersubjective motivational system and the affect regulating functions of the attachment motivational system.

## Introduction

In 1975, Colwyn Trevarthen first presented his groundbreaking explorations of the origins of human intersubjectivity. In the subsequent five decades, his ongoing studies continue to confirm, elaborate, and expand upon these pioneering efforts and to make an enduring contribution to our understanding of early human development. Anchored in what has now become a large body of studies in developmental neuroscience, the central organizing principle of the theory dictates that, from the very beginnings of life, the infant is receptive to and aware of the subjective states of others, particularly the primary attachment object, the mother. This adaptive ability of the infant to bidirectionally communicate its affective states is especially activated in the moments of intimate dyadic free play. His seminal work, confirmed by other major developmental researchers, demonstrated that this capacity for primary intersubjectivity specifically emerges at 2–3 months. At 8 weeks babies are ready to engage in behavioral turn-taking when they expect social contingency, which consists of predictable back-and-forth interactivity. In such face-to-face, eye-to-eye intersubjective emotional communications, the infant and mother, intently looking and listening to each other, synchronize and mutually regulate their emotional states. Indeed, during these protoconversations, the emotions of both members of the dyad are expressed and actively perceived in spontaneous, reciprocal, and rhythmic-turn-taking interactions (Trevarthen, 1993).

**Figure 1:**
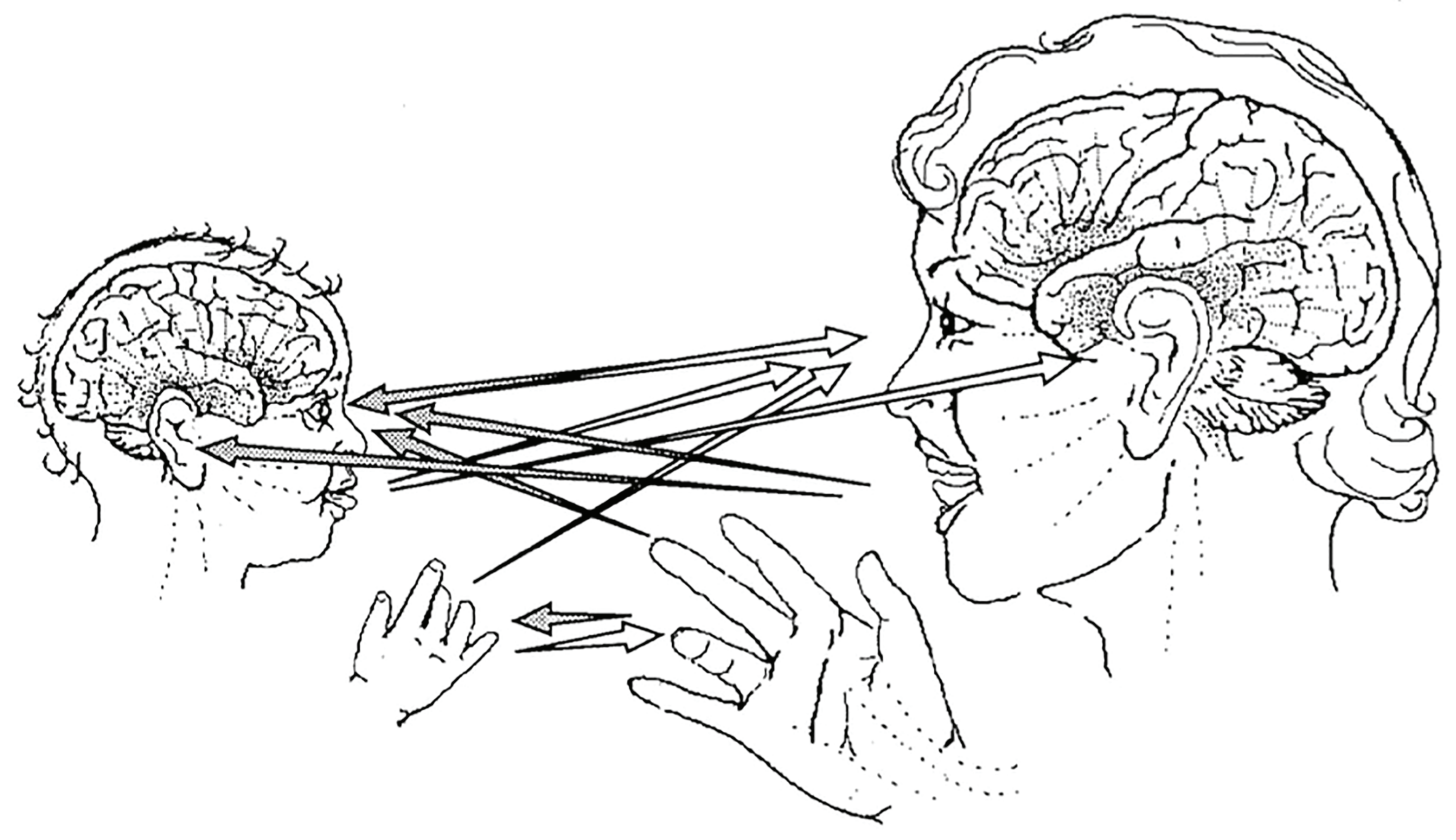
Channels of face-to-face communication in primary intersubjectivity. Protoconversation is mediated by synchronized intersubjective eye-to-eye orientations, vocalizations, hand gestures, and movements of the arms and head, all acting in coordination to express interpersonal awareness and emotions. From Trevarthen (1993).

Within this relational context of primary intersubjectivity, the baby, attracted by the mother’s voice, face expressions, and hand gestures, replies playfully with affection, imitating and provoking imitations. In the same moment, the mother attentively watches and listens, anticipating the baby’s expressions intuitively, and sympathetically replies to the infant’s communications with emotional facial expression, prosodic motherese, and emotional touch. Thus, in this protoconversation of synchronized and coordinated visual facial, auditory, and tactile emotional signals, the mother-infant dyad co-creates an intersubjective reciprocal system of nonverbal communication (see Figure 1). Trevarthen concluded that “The emotions constitute a time-space field of intrinsic brain states of mental and behavioral vitality that are signaled for communication to other subjects and that are open to immediate influence from the signals of these others” (Trevarthen, 1993, p. 155).

Trevarthen (1990, p. 357) observed that this synchronized two-way traffic of reciprocal nonverbal signals elicits instant emotional effects, namely the positive effects of joyful pleasure and excitement build within the emotion transacting protoconversation. But his synchronization model also focused on internal structure-function events, as he stated “the intrinsic regulators of human brain growth in a child are specifically adapted to be coupled, by emotional communication, to the regulators of adult brains.” These regulated intersubjective interactions permit the intercoordination of positive affective brain states within the emotionally communicating dyad. His work underscored the fundamental principle that the baby’s brain is not only affected by these relational emotional transactions, but also its growth literally requires brain-brain interaction in the context of a burgeoning positive affective *relationship* between the mother and her infant. This fundamental interactive mechanism requires older brains to engage with mental states of awareness, emotion, and interest in younger brains and involves coordination between the subjective feelings of an adult and the intersubjective motivations of the infant to forge an emotional bond with the mother.

Trevarthen (1990, p. 335) emphasized the critical role of interpersonal resonance in these intersubjective communications:

Corresponding generative parameters in…two subjects enable them to resonate with or reflect on one another as minds in expressive bodies. This action pattern can become ‘entrained,’ and their experiences can be brought into register and imitated. These are the features that make possible the kind of affectionate empathic communication that occurs, for instance, between young infants and their mothers (1993, p. 126).

Furthermore, he observed “Adaptation of a given brain to a particular social world depends…on a motivated search by the young for certain target experiences (as in) expressing mental or motivational states to others, and getting into contact with their mental states.” In this dyadic state of interpersonal resonance, the infant is “able to exhibit to others at least the rudiments of individual consciousness and intentionality” (Trevarthen and Aitken, 2001, p. 5). Thus, this two-person interpersonal context of primary intersubjectivity also serves as a developmental origin of not only subjectivity but also “self-consciousness.”

In this work, I shall offer an interpersonal neurobiological model of Trevarthen’s intersubjective protoconversations between the mother and her 2–3-month-old preverbal infant. Following the article outline in the abstract, in the upcoming sections, I will offer an operational definition of intersubjectivity as rapid, reciprocal, bidirectional right brain-to-right brain visual-facial, auditory-prosodic, and tactile-gestural positively valenced nonverbal communications between the mother and her developing infant. Expanding this model, I then discuss the fundamental role of interpersonal synchrony in intersubjective protoconversations, as well as how these right-lateralized emotional transmissions generate bioenergetic positively charged interbrain synchrony within the dyad. I then cite recent brain laterality research on the essential functions of the right temporoparietal junction, a central node of the social brain, in face-to-face nonverbal communications. In the following section, I discuss the continued development of right brain intersubjectivity in the 2nd year and beyond, and the relational origins of the highly adaptive functions of mutual play and mutual love. In the final section, I offer thoughts on the clinical applications of Trevarthen’s intersubjectivity and protoconversations in psychotherapy, offering very recent hyperscanning research that demonstrates a right brain-to-right brain nonverbal emotional communication system embedded in the co-constructed therapeutic relationship. I end by offering thoughts on the relationship between the affect communicating functions of the intersubjective motivational system and the affect regulating functions of the attachment motivational system.

## Regulation Theory Models Intersubjectivity as Right-Lateralized Nonverbal Emotional Communications

In my own studies on the early development of intersubjective nonverbal emotional communication, I have utilized the interdisciplinary perspectives of interpersonal neurobiology and regulation theory, a theory of the development, psychopathogenesis, and treatment of the subjective self (Schore, 1994/2016, 2003a,b, 2012a, 2019a,b). The central focus of this psychoneurobiological model of human development is to more deeply understand the underlying mechanisms by which the structure and function of the mind and brain are shaped by experiences, especially those embedded in emotional relationships, as well as the relational mechanisms by which communicating brains synchronize and align their neural activities with other brains. With respect to this nonverbal communication between brains, I have drawn upon the overlap of Trevarthen’s work on intersubjectivity and the Bowlby’s on attachment theory. Although the former focused on emotion transacting events early in the 1st year and the latter on emotional events late in the 1st and 2nd year, both offered a similar model of nonverbal visual-facial, auditory-prosodic, and tactile-gestural communications between mother and infant.

In a mirror image of Trevathen’s two-way traffic of emotional facial expressions, gestures, and vocal expressions, Bowlby (1969, p. 120) proposed that mother-infant attachment communications are “accompanied by the strongest of feelings and emotions, and occur within a context of facial expression, posture, and tone of voice.” Interestingly, as opposed to Bowlby, Trevarthen’s research was directly informed by extensive studies of developmental brain laterality (see his 1996 “Lateral Asymmetries in Infancy: Implications for the Development of the Hemispheres”). Indeed, in that publication, he noted that the prosody of the voice of the mother is responded to by the infant’s right hemisphere. He also concluded that “The right hemisphere is more advanced than the left in surface features from about the 25th (gestational) week and this advance persists until the left hemisphere shows a post-natal growth spurt starting in the second year” (Trevarthen, 1996, p. 582).

Following these valuable leads, in my first book *Affect Regulation and the Origin of the Self* (1994), I drew upon a large body of research on brain laterality and hemispheric asymmetries of structure and function to describe the intersubjective protoconversation as a right-lateralized, reciprocal, and nonverbal emotion communication system. Toward that end, I cited a large number of extant researchers who offered evidence on the early development of right hemisphere (see Schore, 1994/2016 for references) and concluded that the essential adaptive capacity of intersubjectivity is specifically impacted by the infant’s early social experiences. Since these social interactions are occurring in a critical period of right brain growth, the child is using the output of the mother’s right cortex as a template for the imprinting, the hard wiring of circuits in his own developing right cortex that will come to mediate his expanding social-emotional capacities to appraise variations in both external and internal information. I further proposed that, over the course of human infancy, these right brain-to-right brain nonverbal affective communications represent a relational context in which the primary caregiver psychobiologically attunes to and regulates the infant’s internal states of autonomic nervous system and central nervous system arousal. Although Trevarthen stressed the role of intersubjectivity in positively charged play states, my work also addressed the nonverbal intersubjective communications of negatively valenced emotional states between the infant’s mind/body and the mother’s mind/body.

More recently, I have suggested that intersubjective mother-infant nonverbal communications directly influence the “early life programming of hemispheric lateralization” (Stevenson et al., 2008, p. 852) and are a major contributor to dominance of the right brain in human infancy (Schore, 1994/2016; Chiron et al., 1997). Neuroscientists are now asserting that one measure of healthy development in infants is lateralized behavior (Hall et al., 2008). A large body of laterality research in developmental neuroscience demonstrates the adaptive role of the infant’s early maturing right brain in processing visual-facial, auditory-prosodic, and tactile-gestural nonverbal communications (Schore, 2003a, 2012a, 2019a). Indeed, over all stages of human development, “The neural substrates of the perception of voices, faces, gestures, smells, and pheromones, as evidenced by modern neuroimaging techniques, are characterized by a general right-hemispheric functional asymmetry” (Brancucci et al., 2009, p. 895).

With respect to *visual-facial nonverbal communications*, it is now established that mutual gaze is essential for early social development (Trevarthen and Aitken, 2001). The development of the capacity to efficiently process information from faces requires visual input to the right (and not left) hemisphere during infancy (LeGrand et al., 2003). At 2–3 months of age infants show right hemispheric activation when exposed to a woman’s face (Tzourio-Mazoyer et al., 2002). By 6 months, infants express a right-lateralized, left gaze bias when viewing faces (Guo et al., 2009) and significantly greater right frontotemporal activation when viewing their own mother’s (as opposed to a stranger’s) face (Carlsson et al., 2008). On the other side of the mother-infant dyad, a large body of adult research indicates that the right occipital-temporal cortex generates a holistic face representation at 170 ms after stimulus onset, beneath conscious awareness (e.g., Jacques and Rossion, 2009).

Ongoing developmental neurobiological studies of *auditory-prosodic nonverbal communications* reveal that maternal infant-directed speech (“motherese”) activates the right temporal area of 4–6-month-old infants, and that this activation is even greater in 7–9-month-old infants (Naoi et al., 2011). Seven-month-old infants respond to emotional voices in a voice-sensitive region of the right superior temporal sulcus, and happy prosody specifically activates the right inferior frontal cortex (Grossmann et al., 2010). These authors conclude that “The pattern of findings suggests that temporal regions specialize in processing voices very early in development and that, already in infancy, emotions differentially modulate voice processing in the right hemisphere” (2010, p. 852). As to the mother’s emotional prosodic participation very recent adult research demonstrates a “right-lateralized unconscious, but not conscious processing of affective environmental sounds” (Schepman et al., 2016, p. 606).

With respect to *tactile-gestural nonverbal communications*, Sieratzki and Woll (1996) describe the effects of touch on the developing right hemisphere and assert that the emotional impact of touch is more direct and immediate if an infant is held to the left side of the body (see the studies of “left sided cradling” and activation of the right hemisphere in mother and infant in Schore, 2012a, 2019a). Nagy (2006, p. 227) documents a “lateralized system for neonatal imitation” and concludes that “The early advantage of the right hemisphere in the first few months of life may affect the lateralized appearance of the first imitative gestures.” Developmental research demonstrates the essential role of maternal “affective touch” on human infant development in the 1st year of life (Ferber et al., 2008). This allows the infant and mother to create a system of “touch synchrony” in order to alter vagal tone and cortisol reactivity (Feldman et al., 2010). The dyad thus uses “interpersonal touch” as a communication system, especially for the communication and regulation of emotional information.

In order to process these intersubjective nonverbal communications, the infant seeks proximity to the mother, not just physical proximity but intersubjective emotional proximity, face-to-face, mind-to-mind, and body-to-body communications. During these nonverbal communications, the sensitive primary caregiver’s right brain implicitly (unconsciously) attends to, perceives, recognizes, appraises, and regulates nonverbal expressions of the infant’s more-and-more intense states of positive and negative affective arousal. The temporal dynamics of these intersubjective, rapid, spontaneous, and bodily-based right brain nonverbal communications are described by Lyons-Ruth, who observes that implicit, nonconscious processing of nonverbal affective cues in infancy “is repetitive, automatic, provides quick categorization and decision-making, and operates outside the realm of focal attention and verbalized experience” (1999, p. 576). Lyons-Ruth (1999) characterizes a “two-person unconscious” in the intersubjective dialog. From an interpersonal neurobiological perspective, intersubjectivity represents a co-created system of unconscious communications of positive and negative affect between two subjective minds, throughout the life span. My ongoing studies in the field of neuropsychoanalysis, the neuroscience of unconscious processes, continues to offer interdisciplinary evidence showing that the right brain, the psychobiological substrate of the human unconscious mind, acts as a relational unconscious that communicates with another relational unconscious (e.g., Schore, 2003b, 2011, 2012a, 2019b).

## Right-Lateralized Interpersonal Synchrony in Face-to-Face Protoconversations

In this section, I would like to return to Trevarthen’s groundbreaking descriptions of infant-mother synchrony in face-to-face protoconversations. Aitken and Trevarthen (1997) asserted that

In interaction between a normal infant and a happy and receptive caregiving companion the dual intrinsic motive formation systems of the two subjects are mutually supportive in rhythmic, sympathetic engagements which demonstrate *synchrony and turn-taking* in utterances and clear flexible emotionally toned phrasing with affect attunement (p. 667, italics added).

These authors documented the critical role of facial movements, voice, and gesture used by infants in their synchronized engagement with mothers. The timing and organization of playful events between infants and mothers allow the child to adaptively *synchronize* their *subjective* states of mind so that purposes, interests, and feelings are shared, *intersubjectively*. Indeed, interpersonal synchrony is a central construct that lies at the core of Trevarthen’s right brain-to-right brain intersubjective protoconversation.

In this same time period, parallel studies using simultaneous two camera videotape recordings of the mother-infant interaction confirmed the centrality of interpersonal synchrony: Ed Tronick and Berry Brazelton (Tronick et al., 1977), Beatrice Beebe and Dan Stern (Jaffe et al., 2001), and Dorothy Feldman (Feldman et al., 1999). These latter authors were exploring moments of “affect synchrony” that occur in dyadic positive affectively charged social play, clearly reflecting Trevarthen’s primary intersubjectivity:

Face-to-face interactions, emerging at approximately 2 months of age, are highly arousing, affect-laden, short interpersonal events that expose infants to high levels of cognitive and social information. To regulate the intensity of their affective behavior within lags of split seconds (Feldman et al., 1999, p. 223).

Feldman et al. observed that, in this infant-leads-mother-follows sequence effect, synchrony affords infants “their first opportunity to practice interpersonal coordination of biological rhythms, to experience the mutual regulation of positive arousal, and to build the lead-lag structure of adult communication” (p. 223). Furthermore, they asserted that “Synchrony in dynamic systems…reflects the degree to which interactants *integrate* into the flow of behavior the ongoing responses of their partner and the changing inputs of the environment” (p. 224, italics added).

Over 25 years ago, in my first book *Affect Regulation and the Origin of the Self*, I cited the classic research of Lester et al. (1985) who asserted that “synchrony develops as a consequence of each partner’s learning the rhythmic structure of the other and modifying his or her own behavior to fit that structure” (p. 24). The word “synchrony” derives from the Greek words *syn*, which means the same or common, and *chronos*, which means time, and so “synchrony” literally means “occurring at the same time.” Across literatures the construct of synchrony is tightly associated with affective reciprocal interchange, emotion transmission, physiological linkage, and coregulation, all aspects of an intersubjective protoconversation. In a reciprocal, turn-taking communication system both individuals align, synchronize, and match their psychobiological states and then simultaneously adjust their social attention, stimulation, and accelerating arousal to each other. This synchronization occurs at different levels, from neural activity, to physiological states, such as heartbeat rhythm, to pupil size, to facial expressions and body postures (see Schore, 2019a, for references).

A large body of developmental research now documents mother-infant physiological synchrony at 3 months (Moore and Calkins, 2004), 6 months (Moore et al., 2009), and 12 months (Ham and Tronick, 2006) of age, a period when the mother-infant nonverbal affective protoconversations become more complex. Feldman’s laboratory shows that mother and infant coordinate autonomic heart rhythms in moments of interaction synchrony (Feldman et al., 2011). These studies describe the longitudinal development of the capacity for synchronized intersubjective communications between the mother’s mind/body and the infant’s developing mind/body, as well as the enduing impact of early emotional communications on the adaptive capacity for intersubjectivity over later stages of human development. Indeed, mother-child behavioral synchrony is individually stable from infancy through adolescence (Feldman, 2010). Interestingly, in a recent study of what Feldman now terms “social synchrony” in mother-child dyads, she is calling for a “move from focus on one-brain functioning to understanding how two brains dynamically coordinate during real-life social interactions” (Levy et al., 2017, p. 1036) and, in other words, research on *interbrain synchronization*. Note that interpersonal synchrony refers to a synchronization of subjective states, involuntary behaviors, and physiological rhythms between the minds and bodies of two individuals, while interbrain synchrony refers to an alignment of brains between two individuals. Over four decades, my work on *right brain-to-right brain nonverbal communication* describes the right-lateralized interbrain synchronization embedded in the mother-infant (and therapist-patient) relationship.

In my ongoing writings, I continue to offer an interpersonal neurobiological model of the ontogeny of intersubjectivity over the 1st years of human life (e.g., Schore, 2012a, 2019a, 2019b). The early substratum of this adaptive capacity is laid down in the *prenatal period* and in the mutual regulating relationship between the fetus’ and mother’s physiological systems across the placenta. In the last trimester of pregnancy, this dyadic system is centrally involved in the fetal programing of the stress regulating hypothalamic-pituitary-adrenal axis. At this point in development, the paraventricular and ventromedial areas of the hypothalamus are activated in stress regulation, the right insula onsets its stress-responsive visceroautonomic functions, and the regulatory functions of the central and medial functions of the amygdala and their dense connections into the autonomic nervous system, come on line (Schore, 2017a).

During this same time, frame developing structures in the fetal brain support a critical period of growth of the rapidly maturing autonomic nervous system, what Jackson (1931) described as “the physiological bottom of the mind.” Porges (2011) offers research evidence documenting that the early forming, oldest, parasympathetic unmyelinated dorsal nucleus of the vagus, the later developing catecholaminergic sympathetic nervous system, and the last developing and newest parasympathetic myelinated ventral vagal system in the nucleus ambiguus are functioning at the start of the last trimester. In discussing “the development of the autonomic nervous system in the human fetus,” he concludes that “The unique features of the autonomic nervous system that support mammalian *social behavior* start to develop during the last trimester of fetal life” (p. 126, italics added). Underscoring the laterality of these ANS subsystems, he proposes a right-lateralized circuit of emotion regulation that supports the functional dominance of the right side of the brain in regulating autonomic function. Porges further states that the maturation of this ventral vagal system continues well into the first year.

In the ensuing perinatal postpartum stage after birth, the psychobiologically attuned mother and the neonate begin to co-create face-to-face communications that are driven by subcortical face processing areas. In body-to-body communications, the infant also processes olfactory stimuli that emanate from the mother’s body. In previous writings, I have offered evidence that, in this earliest stage of postnatal development, a critical role is continued to be played by the central medial amygdala, with its deep connections into bioaminergic arousal centers in the midbrain and brain stem and the sympathetic and parasympathetic components of the autonomic nervous system (Schore, 2014b, 2017a, 2019a). In these primordial nonverbal communications, the mother regulates the infant’s internal states of sympathetic and parasympathetic autonomic arousal, thereby facilitating a burgeoning state of autonomic balance and a subjective sense of safety, expressed in the infant’s quiet alert state (Schore, 1994/2016). In classic writings, Basch (1976) stated that “the language of mother and infant consist of signals produced by the autonomic, *involuntary* nervous system *in both parties*” (1976, p. 766, italics added). Thus, these reciprocal bidirectional autonomic processes are expressed in involuntary and not voluntary motor behavior.

I suggest that the early postnatal stage of human development is a critical period for a transition from subcortical to cortical face processing systems and from the dorsal vagal to the experience-dependent maturation of the right-lateralized ventral vagal social engagement system of Porges (2011). With direct relevance to the precursors of intersubjectivity, Porges asserts “The right vagus and, thus, cardiac vagal tone are associated with processes involving the expression and regulation of motion, emotion, and communication” (p. 140), and that “the vagal control of the right side of the larynx produces changes in vocal intonation [prosody] associated with expression of emotions” (p. 141, italics added). According to Manini et al. (2013), “The autonomic nervous system seems to represent an elementary mechanism supporting emotional synchrony between mother and infant” (2013, p. 2). This maturational advance heralds the onset of mother-infant right hemispheric eye-to-eye, body-to-body left-sided cradling, an evolutionary facilitator of social cognition (Forrester et al., 2018; Schore, 2019a,b).

Indeed, at around 8 weeks, the onset of primary intersubjectivity, there is a dramatic progression of the infant’s social and emotional capacities. This postnatal period is initiated in a critical period of development of the infant’s posterior right cortical areas involved in sensory processing, the right insula and its autonomic connections, the right basolateral amygdala and its dense connections with cortical areas, and the medial frontal areas in the right anterior cingulate associated with responsivity to social cues (Schore, 2019a). Within episodes of mutual gaze, the most intense form of human communication, the intuitive mother’s and infant’s right brains engage in synchronized, spontaneous facial, vocal, and gestural communications of positive emotional states (see Figure 1). Such highly arousing, emotion-laden, and face-to-face interactions allow the infant to be exposed to high levels of social information. In these right brain limbic-autonomic emotional transactions, the mother makes herself contingent, easily predictable, and manipulatable by the infant, and thereby able to interpersonally synchronize her brain with her infant’s developing brain.

Most intriguingly, research documents neuroplastic structural changes in the mother’s brain during this same developmental period (Kim et al., 2010). This longitudinal study included two time points: 2–4 weeks postpartum and 3–4 months postpartum, and therefore over the onset of intersubjectivity, 2–3 months. During this period, gray matter in the mother’s brain increases in specifically her right insula, hypothalamus, anterior cingulate, and amygdala, as well as in the reward-associated mesolimbic dopamine nuclei in the substantia nigra. The authors conclude that interactions with the infant induce these structural changes, which are expressed in functional increases of maternal motivation and sensitivity to infant cues. Indeed, they report “these structural changes at 3–4 months were predicted by a mother’s positive perception of her baby at the first month postpartum. Thus, the mother’s positive feelings for her baby may facilitate the increased levels of gray matter” (Kim et al., 2010, p. 698). This clearly implies that the mother’s positive feelings for her developing baby are associated with subsequent changes in her own brain.

These infant and maternal neurobiological data can be interpreted within the framework of interpersonal neurobiology’s central principles that the structure and function of the mind and brain are shaped by synchronized emotional relationships, and that brains align their neural activities in social interactions. This simultaneous brain growth on both sides of the mother-infant dyad suggests an alignment between mother’s and infants’ right brain cortical-subcortical limbic circuits during positively-valenced intersubjective emotional protoconversations. At 2–3 months, a critical period for the onset of intersubjectivity, the mother’s right basolateral amygdala and anterior cingulate are undergoing neuroplastic reorganization, at the very same time when her infant’s right basolateral amygdala and anterior cingulate are in a critical period of growth. This coordinated accelerated synaptic growth in both brains *occurring at the same time* is another example of mother-infant synchrony defined as coordinated timing in development (Jaffe et al., 2001).

## Intersubjective Bioenergetic Transmissions Generate Positively Charged Interbrain Synchronization

As mentioned, these episodes of “affect synchrony” occur in the first expression of social play. I suggest that these positively charged mother-infant emotional interactions generate increasing levels of dopaminergic arousal, and thereby joy (elation), a state of intense pleasure plus the urge for contact-seeking. Dopamine is the most important catecholamine involved in reward effects, and in this co-creation of a “reciprocal reward system” high levels of ventral tegmental mesolimbic dopamine are generated in both brains. Activation of the mesolimbic dopamine system that exerts a growth-promoting neurotrophic effect on the postnatal cortex is associated with initiation of movements to emotional or motivational stimuli, and the incentive of motivation and anticipation of reward (Schore, 1994/2016). Trevarthen also reported an increased positive state of excitement in protoconversations, which I suggest is associated with states of regulated sympathetic noradrenergic hyperarousal.

Stern (1990) described how infants seek stimulation that arouses, excites, and activates them and find this state of heightened activation intensely pleasurable. He described the energetic capacities of dynamic “vitality affects,” the positive effects that are required to build self-structure and characterized maternal social behavior that can “blast the infant into the next orbit of positive excitation” (Stern, 1985). In such interactions of interpersonal resonance, both partners match states and simultaneously adjust their social attention, stimulation, and accelerating energy-mobilizing catecholaminergic sympathetic autonomic arousal to each other’s responses. On a moment-to-moment basis, the empathic caregiver’s sensory stimulation synchronizes with the crescendos and decrescendos of the infant’s endogenous rhythms, allowing the mother to appraise the nonverbal expressions of her infant’s internal emotional arousal and positive psychobiological states, to mutually upregulate them, and to communicate and intersubjectively share them with the infant.

In these essential face-to-face emotional transactions of mutual gaze, the mother initially attunes to and resonates with the infant’s resting state but, as this state is dynamically activated (or deactivated or hyperactivated), she contingently fine tunes and corrects the intensity and duration of her affective stimulation in order to maintain the child’s positive affect state. As a result of this moment-by-moment synchronized matching of affective direction both partners increase together their degree of engagement and facially expressed positive effect. This interactive microregulation continues, as soon after the “heightened affective moment” of accelerating arousal and an intensely joyful full gape smile the baby gaze averts in order to autoregulate the potentially disorganizing effect of the accelerating arousal of the intensifying emotional state. In order to maintain the positive emotion, the psychobiologically attuned mother takes her cue and backs off to reduce her stimulation. She then waits for the baby’s signals for reengagement, signaled in the reappearance of the infant’s quiet alert state (Schore, 2003a).

In this manner, not only the tempo of their engagement but also their disengagement and reengagement are coordinated and synchronized. In this process of contingent responsivity, “the more the mother tunes her activity level to the infant during periods of social engagement, the more she allows him to recover quietly in periods of disengagement, and the more she attends to the child’s reinitiating cues for reengagement, the more synchronized their interaction” (Schore, 1996, p. 61). The psychobiologically attuned sensitive caregiver who is physiologically synchronized with the child thus facilitates the infant’s emotional information processing by adjusting the mode, amount, variability, and timing of the onset and offset of stimulation to the infant’s actual integrative capacities (Schore, 2003a).

In such synchronized, reciprocal, and turn-taking interactions, the mother must be attuned not so much to the child’s overt behavior as to the reflections of the covert, involuntary physiological autonomic rhythms of his or her internal state, enabling the dyad to co-construct a “mutual regulatory systems of arousal” that contains a “positively amplifying circuit affirming both partners” (see Schore, 1994/2016 on “Mirroring gaze transactions and the dyadic amplification of positive affects”). The capacity of the infant to experience increasing levels of positive arousal states is thus amplified and externally regulated by the primary caregiver and depends on her capacity to playfully engage in synchronized emotional exchanges that generate increased positive arousal in herself and her child (see Figures 2.1 and 2.2 in Schore, 2003a).

I would add that this “interpersonal synchrony” is also expressed in right-lateralized “interbrain synchrony,” simultaneous changes of emotional energy within the right brains of both members of the dyad, In terms of self-organization theory, the mutual entrainment of their right brains during moments of affect synchrony triggers an *amplified energy flow*, which allows for a coherence of organization that sustains more complex states of consciousness within both the infant’s and the mother’s right brains. Recall, the assertions of Trevarthen and Aitken (2001) in synchronized primary intersubjective transactions interpersonally resonated positive states of arousal are *amplified*, that this impacts the infant’s capacity to generate “rudiments of individual consciousness,” and that this two-person interpersonal context serves as a developmental origin of not only subjectivity but also “self-consciousness.”

In parallel writings, Tronick et al. (1998) described the co-creation of an expanded “*dyadic state of consciousness*” within the mother-infant dyad, when the emergent state of consciousness becomes more coherently organized and more complex. Tronick hypothesized that “the capacity to create dyadic states of consciousness with another, and the quality of those states, depends in part on the history the individual had in creating these states early in development with his or her mother (and others)” (p. 298–299). He also proposed that dyadically expanded states of consciousness represent an “unconscious force driving social engagement” (1998 p. 296). In earlier works, I suggested that Tronick is describing an expansion of what Edelman (1989) called as “primary consciousness” that relates visceral and emotional information pertaining to the biological self to stored information processing pertaining to outside reality, which is specifically located in the right brain. Similarly, Trevarthen’s “rudiments of individual consciousness” refer to this intersubjective right-lateralized primary consciousness within the infant’s developing “right mind” (Ornstein, 1997). Right brain “primary consciousness” is thus communicated and generated in right brain-to-right brain “primary intersubjectivity.” Note the intersubjective interbrain synchronization between two right-lateralized subjective minds, and its impact upon a cardinal function of a mind, the generation of states of consciousness.

Furthermore, Feldman et al. (2011) highlighted the relational context of “intense moments” of “interaction synchrony” co-created by 3-month old infants and their mothers:

Face-to-face exchanges are short events spread across the daily routine of parent and child that mark purely social moments and involve *higher levels of positive arousal and social coordination*, as compared to episodes of caregiving and feeding. The brevity and intensity of such moments appear to initiate a process of biological concordance between the partners’ heart rhythms. As seen, during episodes of high positive arousal – for instance, moments of vocal or affective synchrony which are accompanied by *high positive energy* – *the tightness of the biological synchronicity increased*…gaze synchrony in of itself…without a rise in positive arousal, did not increase biological synchrony (p. 573, italics added).

These emotional transactions involving synchronized ordered patterns of energy transmissions (directed flows of energy) represent the fundamental core of the right brain systems of communication and regulation (see Schore, 2003a, 2012a). Synchrony in dynamic systems, including social systems, is viewed as a complex, emergent, and indeterminate process. A central tenet of dynamic systems theory holds that at particular critical moments, a flow of energy allows the components of a self-organizing system to become increasingly interconnected, and in this manner organismic form is constructed in developmental processes. As the patterns of relations among the components of a self-organizing system become increasingly interconnected and well-ordered, it is more capable of maintaining a coherence of organization in relation to variations in the environment. This description applies to the interconnectivity of the cortical-subcortical components of the early developing right hemispheric subjective self-system.

More specifically, in right brain-to-right brain emotion-transacting intersubjective communications, organized patterns of information emanating from the caregiver’s face, voice, and gestures trigger synchronous metabolic energy shifts in the infant’s brain and body, central nervous system, and autonomic nervous system. The caregiver is thus modulating changes in the child’s energetic state, since physiological arousal levels are known to be associated with changes in cellular metabolic energy. Within a co-created intersubjective field, these regulated emotional exchanges *between* the mother and infant in turn elicit synchronized increased energy shifts in both of their right brains. This right-lateralized interbrain synchronization generates dynamic vitality affects in a *co-created energized intersubjective field* also creates metabolic (mitochondrial) biological energy that facilitates the growth and developmental organization *within* the infant’s rapidly growing brain, especially during critical periods of right brain development associated with a regional transition from anaerobic to aerobic metabolism (see “synchronized bioenergetic transmissions” in Schore, 1994/2016). This metabolic energy is imprinted into circuits of synaptic connectivity between the cortical and subcortical levels of the infant’s developing right brain, allowing the emotion processing right brain to act as a dynamical system, a cohesively organized self-regulating *integrated whole*. In this manner, “the self-organization of the developing brain occurs in the context of a relationship with another self, another brain” (Schore, 1996, p. 60). This fundamental intersubjective mechanism of human development lies at the core of what Stern (1977) called as “the first relationship,” and Brazelton and Cramer (1990) termed as “the earliest relationship” between the infant and another human being, the mother.

Indeed, throughout the life span energy shifts are the most basic and fundamental features of emotion, discontinuous states are experienced as affect responses, and nonlinear psychic bifurcations are manifest as rapid affective shifts. Such state transitions result from the activation of synchronized bioenergetic processes in central nervous system cortical and limbic circuits that are associated with concomitant homeostatic adjustments within the autonomic nervous system’s catabolic energy-mobilizing sympathetic and anabolic energy-conserving parasympathetic branches. Furthermore, interpersonal physiological synchrony is expressed in the coupling of the sympathetic and parasympathetic components of the autonomic nervous systems *between* individuals. Physiologically synchronized and mutually regulated emotional mind-body states thus reflect the nonlinear pulsing of positively charged energy flows *within* the components of a dynamic, self-organizing right-lateralized mind-body system of the subjective self, as well as *between* one right-lateralized intersubjective self and another intersubjective self.

## Right Temporoparietal Cortex: a Central Node of the Social Brain

In 1997, I published an article on the organization of the early developing right brain, in which I described the rapid, implicit, and nonconscious emotional energy-dependent imprinting of regional cortical-and subcortical circuits during critical periods of infancy (Schore, 1997). Subsequent research confirmed the early development of the right brain, before the left (e.g., Gupta et al., 2005; Sun et al., 2005; Mento et al., 2010; Ratnarajah et al., 2013). Supporting the idea of an early period of accelerated growth at 2–3 months, research indicates that in the first 3 months brain growth increases by 64% (Holland et al., 2014), and that the total number of cortical neurons in the human brain increases by 23–30% from birth to 3 months (Shankle et al., 1999). In light of the well-documented observation that the onset of primary intersubjectivity occurs at 2–3 months, specifically what cortical right brain structures are in a critical period of growth and synaptic connectivity at this time? Since the infant’s visual-facial, auditory prosodic, and tactile-gestural sensory processing occurs in the posterior cortical areas of the early developing right hemisphere, this right-lateralized posterior cortical region is such a candidate.

In previous writings, I reported extant developmental neurobiological studies of the infant brain showing that regional differences in the time course of cortical synaptogenesis exist, and that the metabolic activity that underlies regional cerebral function is ontogenetically highest in the posterior sensorimotor cortex and only later rises in anterior cortex (Schore, 1994/2016). In the first months of life association, areas of the posterior parietal (somatosensory) cortex mature as a result of high levels of *tactile* bodily sensation provided by the maternal environment, with visual input secondary (Chugani et al., 1987). Yamada et al. (1997) functional magnetic imaging (fMRI) research demonstrated a milestone for normal development of the infant brain occurs at about 8 weeks. At this point, in time a rapid metabolic change occurs in the primary visual cortex of infants. These authors interpret this rise to reflect the onset of a critical period during which synaptic connections in the occipital cortex are modified by *visual* experience. A more recent study documented a large, robust cerebral asymmetry in the infant right superior temporal cortex at 3 months (Glasel et al., 2011). These researchers suggest that this rapid growth is specifically associated with visual processing, voice perception, and nonverbal social communication.

With respect to the processing of *auditory* prosody, a near-infrared spectroscopy (NIRs) study by Homae et al. (2006) revealed that prosodic processing of a female emotional voice occurs in 3-month-old infants, specifically in the right temporoparietal region. Indeed, auditory information emanating from the mother’s face, embedded in the affective tone of her emotionally expressive voice, is known to be processed in the right temporoparietal cortex (Ross, 1983). In discussing the mother-infant interaction, Dissanayake (2017) emphasizes how much all three sensory modalities or “languages” of the intersubjective engagement, facial, vocal, and body, are *processed as a whole* in the infant’s brain during these interactions. The right temporoparietal junction (right TPJ) is known to be activated in the experiencing of *positive affect* associated with *synchronous multisensory stimulation* (Tsakiris et al., 2008).

This right-lateralized system, a heteromodal association area located at the intersection of the *posterior* end of the superior *temporal* sulcus, the inferior *parietal*, and the lateral *occipital* cortex *integrates* these three sensory modalities (the *voice*, and *touch* and *face* of the mother). Indeed, the right temporoparietal system *integrates* input from auditory, visual, somesthetic, and emotional limbic areas and forges critical period connections with the right ventral anterior cingulate involved in responsivity to social cues and play behavior, and the right insula with its extensive connections into the ANS that generates a representation of visceral responses accessible to awareness, thereby providing a somatosensory substrate for subjective bodily-based emotional states experienced by the corporeal self. The right TPJ forges direct synaptic contacts with the right basolateral amygdala and its extensive connections with cortical association areas, and the right-lateralized hypothalamico-pituitary-adrenocortical and sympathoadrenomedullary systems involved in autonomic activity.

During the early postnatal critical period of posterior cortical development, the right TPJ cortical-subcortical system also increases its reciprocal connections with the right ventral striatum and right ventral tegmental areas involved in mesolimbic dopaminergic positive arousal and reward, the right locus coeruleus that generates states of noradrenergic arousal and attention, and the right raphe nucleus associated with the serotonergic modulation of emotional and sensory reactivity (see Schore, 2019a,b). These three bioaminergic arousal-generating systems in the midbrain and brainstem continue to evolve in postnatal periods when they send axon collaterals up the neuraxis, thereby exerting trophic, energetic, and regulatory roles on the development of the cerebral cortex and limbic system (Schore, 2012a, 2019a). In total, this increased cortical-subcortical interconnectivity of the right TPJ cortical-subcortical sensoriaffective system allows for the infant’s developing right brain to form more complex implicit visual-facial, auditory-prosodic, and tactile-gestural nonverbal communications, and thereby even greater capacities for intersubjectivity.

A large body of recent research now indicates that the functions of the right TPJ strikingly mirror the central functions of Trevarthen’s primary intersubjectivity. Recall his description of the interpersonal context of protoconversation: face-to-face, interactively synchronized, reciprocal, and rhythmic-turn-taking social interactions embedded in an intersubjective nonverbal communication system that evolves in intimate free play and co-creates a positive affective relationship and interpersonal awareness between mother and infant (see Figure 1). Researchers are emphasizing “the importance of the right temporoparietal junction in collaborative social interactions” (Tang et al., 2016, p. 23) and are documenting its fundamental involvement in the building of positive relationships (Kinreich et al., 2017).

Interdisciplinary studies now document that this posterior right cortex, a central node of the social brain (Santiestaban et al., 2012), is activated in face-to-face transactions (Redcay et al., 2010), where it functions in “attention and social interaction” (Krall et al., 2015, p. 587) in a social context of a “basic interpersonal interaction” (Goldstein et al., 2018, p. 2532). The right TPJ serves as a convergence zone of sensory and contextual information, which is then integrated to create a social context with other social agents (Lee and McCarthy, 2016). This multifunctional system is centrally involved in updating one’s internal model of the current environment (i.e., contextual updating) and adjusting expectations based on incoming sensory information (Geng and Vossel, 2013). It responds to visual, auditory, and tactile stimuli (Matsuhashi et al., 2004) and is specialized for the detection of personally relevant stimuli, particularly when they are salient or unexpected (Corbetta and Shulman, 2002).

Furthermore, authors are now asserting that this right hemispheric temporoparietal polysensory area “plays a key role in perception and awareness” (Papeo et al., 2010, p. 129) and, in “the unconscious guidance of attention,” “*outside of conscious awareness*” (Chelazzi et al., 2018, p. 2, italics added). This right-lateralized implicit system is involved in “the control of self-and other representations” (Santiestaban et al., 2012, p. 2274) and in the ability to “switch between internal, bodily, or self-perspective and external, environmental, or other’s viewpoint” (Corbetta et al., 2008, p. 317). This posterior area of the right hemisphere is also a pivotal neural locus for multisensory body-related information processing (Decety and Lamm, 2007, p. 580), and for “maintaining a coherent sense of one’s body” and a “subjective feeling of body ownership” (Tsakiris et al., 2008). These latter authors observe that this structure generates “an *internal model of the body* that would function as a stored template against which to compare *novel* stimuli, playing a role in maintaining a basic sense of *embodied self*” (p. 3015, italics added).

In seminal studies, Decety and Lamm (2007) wrote on the central role of the right temporoparietal junction in *social interaction and self-functions*, and in another Decety and Chaminade (2003) concluded that “self-awareness, empathy, identification with others, and more generally intersubjective processes are largely dependent upon…right hemisphere resources, which are the first to develop” (p. 591). Indeed, this right-lateralized system is known to be fundamentally involved in face and voice processing, as well as “making sense of another mind” (Saxe and Wexler, 2005, p. 1391). The dynamic intersubjective interaction of one right temporoparietal system with another right temporoparietal system in a collaborative social interaction is thus expressed in a face-to-face right brain-to-right brain nonverbal communication embedded in an intersubjective protoconversation.

Note the remarkable complexity of the subjective and intersubjective functions of the right TPJ cortical-subcortical system in the early postnatal period. As mentioned these adaptive, indeed essential primordial psychobiological functions include a developing ability to engage in nonverbal emotional communications with another human being and share a positive emotional state, a responsiveness to social relational cues, a capacity to integrate sensoriaffective stimuli, and the detection of personally relevant stimuli particularly when they are salient or unexpected, as well as perception, attention, awareness, consciousness, and a representation of an embodied self. These recent discoveries in neuroscience underscore the central importance of this right-lateralized system in human development. Yet, I suggest that this early appearing psychic structure that *operates outside of conscious awareness* has previously extensively described by Sigmund Freud, and that its adaptive functions lie at the core of psychoanalytic theory.

Utilizing a neuropsychoanalytic perspective, I deduce that the multifunctional right temporoparietal system is isomorphic with Freud’s early developing unconscious corporeal ego. In *The Ego and the Id*, Freud (1923/1961) concluded that “The ego is first and foremost a *bodily ego*.” Freud spoke of an early developing unconscious ego and a later developing conscious ego, which “is in control of voluntary movement” and is “located in the speech area on the left-hand side” (Freud, 1923/1961), clearly alluding to the posterior regions of the left hemisphere and Wernicke’s receptive language area, and what neuroscientists are now describing as a “verbally driven ego-bound mode of ordinary consciousness” (Flor-Henry et al., 2017). Yet, Freud also stated “A part of the ego – and heaven knows how important a part – may be unconscious, undoubtedly is unconscious” (Freud, 1923/1961). He further asserted “The processes in the Ego (they alone) may become conscious. But they are not all conscious, nor always so, nor necessarily so; and large parts of the Ego may remain unconscious indefinitely” (Freud, 1926/1961).

These neuropsychoanalytic data suggest that the early developing coherently organized unconscious ego is neuroanatomically located in the right brain, the psychobiological substrate of the unconscious mind. They indicate a significant modification of Freud’s theory – this right posterior cortical-subcortical system is involved in not only intrapsychic but also interpersonal functions of a relational unconscious that intersubjectively communicates with another relational unconscious. In optimal interpersonal contexts, at 2–3 months the human bodily-based unconscious ego can nonverbally communicate with another unconscious ego *via* intersubjective, synchronized, and reciprocal right temporoparietal-to-right temporoparietal social-emotional interactions. Furthermore, science now documents that the earliest expression of the human unconscious mind is not a Freudian intrapsychic cauldron of untamed passions and destructive wishes but an interpersonal generator of amplified joy and shared love. On the other hand, this research confirms Freud’s fundamental discoveries, demonstrating that this right-lateralized system of social connectedness, a deep core of the personality, operates implicitly, beneath awareness as an unconscious ego in everyday life, across the life span. These findings underscore the fact that the human unconscious, a central construct of psychodynamic theory and clinical practice for the past 100 years, needs to be reinserted into academic developmental psychology from which it has almost been banished.

## The Development of Intersubjectivity Over the 1st Year and Beyond

During the 2–3 month transitional period of the human brain growth spurt, new adaptive functional advances of the rapidly maturing right brain emerge, including the capacity to intersubjectively emotionally communicate with other minds, and the ability to share intense emotional states with another human being. In classic developmental research, the psychoanalyst-psychiatrist Stern (1985) described the transition from an early forming “emergent self” at birth into a “*core self*” at 2–3 months, the exact interval of Trevarthen’s primary intersubjectivity. He observed, “At the age of 2–3 months, infants begin to give the impression of being quite different persons. When engaged in social interaction, they appear to be more wholly *integrated*. It is as if their actions, plans, affects, perceptions, and cognitions can now all be brought into play and focused, for a while, on an interpersonal situation” (p. 69, italics added). Stern noted that with the onset of this emergent developmental function, the subjective social world is altered and interpersonal experience operates in a different domain, a domain of “core-relatedness.” He concluded that at this developmental stage, the infant participates in shared “observable interactive events” involved in “bridging the infant’s subjective world and the mother’s subjective world” (p. 119), and that now “There are many ways being with an other can be experienced…such as merging, fusion…symbiotic states” (p. 100). I suggest that these early evolving intersubjective functions of the core self are associated with the infant’s developing right temporoparietal self-system,

In their research on the 2–3 month transition, Ammaniti and Gallese (2014) reported that

From the second month after birth, parents and infant begin to show a temporal structure in their interactions… In this period, the sharing of social gaze between parent and baby is the expression of coordinated [synchronized] interactions, which can occur between 30% and 50% of the time. At the same time, mutual gaze can be integrated with parents’ and infants’ affectionate touch… At around 3 months, parents tend to touch their baby in an affectionate way and infants tend to respond with an intentional *affectionate touch* (p. 147, italics added).

Note the increases of the mother’s loving touch that emerge at this time period.

Confirming this same transitional critical period Miall and Dissanayake (2003) documented changes in the mother’s behaviors:

Over time, mothers subtly adjust their sounds and movements to what the baby seems to want (or not want), and to its changing needs and abilities. They gradually move from the gentle, cooing reassurance of the first weeks to trying to engage the baby in increasingly animated *mutual play*. At 8 weeks utterances and facial expressions have become more *exaggerated*, both in time and space (2003, p. 342, italics added).

Dissanayake (2017) also described prosodic motherese as dyadic in nature, where both partners influence each other, and multimodal, where multiple senses are involved. Frame-by-frame microanalyses of videotaped mother-infant interactions showing the faces and torsos of both partners side-by-side reveal that facial expressions and head-and-body movements are as significant in the interaction as vocalizations (Murray and Trevarthen, 1985).

In another study of the co-created positively valenced nonverbal communication system between mother and infant that evolves in this same time period, Dissanayake (2001) asserted that

It should also be emphasized here that although mothers ‘talk’ to their babies, the multimodal messages in early interactions are nonverbal. What mothers convey to infants are not their verbalized observations and opinions about the baby’s looks, actions, and digestion—the ostensible content of talk to babies—but rather positive affiliative messages about their intentions and feelings: You interest me, I like you, I am like you, I like to be with you, You please me, I want to please you, You delight me, I want to communicate with you, I want you to be like me (p. 91).

In the intimate context of *intense positive affect*, these are the first nonverbal communications of maternal love. The Oxford English Dictionary defines love as “*deep* affection, *strong* emotional attachment.”

In very recent writings, I have described how Trevarthen’s primary intersubjectivity evolves in an intimate context of interpersonally synchronized *mutual play*, a shared positive affective relationship that amplifies *intense* joy and excitement, and that this same *intimate* context of maternal affection also interactively generates the *strong emotions* of *mutual love* (Schore, 2019a,b). In those volumes, I suggest that the loving mother’s and infant’s right brain-to-right brain intimate playful nonverbal communications generate high levels of accelerating amplified positive emotional arousal, which fundamentally is known to be associated with changes in metabolic energy. The emotional energy embedded in this intense positive affective state of deep affection of mutual love is available to the infant’s developing right brain while exposed to high levels of spontaneous interpersonal and intrapersonal novelty, allowing for the multimodal integration of external and internal sensations.

In this manner, the energized intersubjective field of mutual love structuralizes Stern’s “core self” that appears at 2–3 months, a critical period of right brain development, and thus the core self, operating at levels beneath conscious awareness, has an enduring influence on the capacity to co-create an emotional loving bond with a valued other at later stages of the life span. Ammaniti and Gallese (2014) offered an evocative portrayal of Stern’s model of the similar interpersonally synchronized expressions of mutual love in early and later development:

As Daniel Stern has written, expressions of love begin early in an astonishing way. Mother and child behavior overlaps with the behavior of two lovers. For example, mother and child look at each other without speaking, hold a physical closeness with faces and bodies in constant contact, display alterations in vocal expressions or *synchrony of movements*, and perform particular gestures like kissing each other, hugging, touching, and taking the face or the hands of the other… When parents speak to their child, or lovers talk with one another…they emphasize the musicality of the words instead of the meaning, they use baby talk, and they express a wide range of nonverbal vocalizations… Facial expressions assume a special register also, altering and emphasizing the facial mimic. There is also a choreography in the movements of mother and baby, like those of two lovers; *they move in synchrony, getting closer and more distant on the basis of a common rhythm* (pp. 110–111, italics added).

For an interpersonal neurobiological model of the capacity for mutual love, I refer the reader to my chapter “The development of the right brain across the life span: what’s love got to do with it?” (Schore, 2019a).

Authors are now asserting that, over the 1st year, “intersubjective behavior continues to grow significantly over the semesters” (Muratori et al., 2011, p. 19). The synchronized right brain-to-right brain mother-infant intimate playful intersubjective protoconversation continues over human infancy in mutual play, songs, lullabies, and nursery rhymes. In peek-a-boo episodes, maternal affect matches, synchronizes, and amplifies infant joy. A mother playing peek-a-boo will delay the removal of her hands from her eyes in order to provoke amusement and laughter from her baby or similarly when reciting “This Little Piggy” will wait to utter what the fifth piggy squeals – “wee, wee, wee, all the way home.” Recall, the right temporoparietal system responds to visual, auditory, and tactile stimuli and the detection of personally relevant stimuli, particularly when they are *salient or unexpected*. In this early mutual play, repetition in the mother’s exaggerated facial expressions, vocal utterances, and body movements and the effect of surprise coordinates and synchronizes the minds and brains of two bodies, mutually regulating the infant emotionally and uniting mother and child temporally (see Schore and Marks-Tarlow, 2018). Thus, over the course of the 1st year, *intersubjective play* occurs in a relational context of what Tronick (2007) terms “mutual regulation.”

The perspective of interpersonal neurobiology suggests that mother-infant play is more socioemotional than cognitive, and that, fundamentally, the underlying mechanism of this arousal-altering, pleasurable, rewarding mutual activity facilitates the experience-dependent maturation of right brain cortical and subcortical systems. This primordial form of intersubjective play generates the neurobiological substrate on which all forms of play evolve – mother-infant and solitary, spontaneous and controlled, and active and passive (see Schore, 2017b interview in the *American Journal of Play*). During the 1st year, intersubjective synchronized mutual play expands the infant’s affect array and facilitates the dyadic amplification and transformation of mildly pleasurable enjoyment into joy and the intensification of mildly pleasurable interest into excitement. At 10–12 months, the onset of a critical period for the right orbitofrontal cortex and the emergence of upright locomotion, fully 90% of maternal physical and verbal behavior consists of affection, play, and caregiving, and by 1 year of age, curiosity and stimulation-seeking exploratory play time increases to as much as 6 h of a child’s day (Schore, 1994/2016).

My colleague Meares (2016, p. 52) asserts “the mother-infant protoconversation represents an interplay between two right brains.” He argues that in optimal developmental contexts, the right brain-to-right brain protoconversation continues in the second year, a time when a toddler develops a burgeoning playful imagination and shows an expanded need for novel experiences. With the ongoing expansion of higher right brain functions, the intersubjective protoconversation takes the form of intersubjective imaginative games, then intrasubjectively internalized dialogs, and finally what Meares calls “conversational play.” This creative game, which the toddler plays while alone, is grounded in the child’s burgeoning capacity for make-believe and is expressed in the expressive use of emotional words and analogy. Indeed, Meares describes it as right hemispheric analogical or protosymbolic play, which is imbued with the affective dimension of joy and pleasure. The creative game consists of a miniature story, told as if to the child himself or herself but also to someone else, who is not there except as a feeling of the background presence of the internalized, protoconversational mother. This earliest form of symbolic play allows the toddler to play with ideas and generate fantasies, including fantasied interactions with other minds (Schore, 2017b). Furthering these ideas, I would add that, upon entering early childhood, these products of the emergent imagination can also be shared with a valued other in the intersubjective play of creative storytelling.

The psychoneurobiological substrates of symbolic play and imagination, heavily influenced by right brain activation, emerge at the end of the human growth spurt, late in the second year (Dobbing and Sands, 1973). These two right-lateralized adaptive functions, plus another, mutual love, are higher right brain functions built upon a shared common lower-level processes, primary intersubjectivity and protoconversation, which onset at the beginning of the right brain critical period. In this manner, the interpersonally synchronized right brain-to-right brain intersubjective communications that begin to structuralize the right temporoparietal core self at 2–3 months represent the primordial developmental crucible of the adaptive capacities of mutual play, love, and imagination (see Schore, 2019a,b). These fundamental expressions of humanness can be accessed not only *within* a mind but also intersubjectively shared *between* human minds.

The right hemisphere continues to enter into growth spurts in the later stages of development (particularly adolescence), and so the right-lateralized synchronized intersubjective communication system can continue to evolve across the life span. Throughout life interpersonal synchrony, operating beneath levels of awareness, acts as a fundamental interpersonal neurobiological mechanism not only within *dyads*, but also in all human *group* dynamics, and in the organization of all *cultures* (Schore, 2012b, 2020). That said it should be pointed out that the well-documented time frame of the primordial critical period for intersubjectivity is not protected in the United States, which is the only industrialized country that has no maternal leave policy, leading many mothers to re-enter the work force at 6 weeks and put the child into day care, before the 2–3 month transition even begins. This lack of legislated temporal protection for the establishment of the foundations of a loving maternal-infant relationship may in turn have long-term effects on the emotional health of the culture, especially on males, whose right brains mature more slowly than females, making them more vulnerable to early relational stressors and susceptible to developmental disorders and externalizing psychopathologies (Schore, 2012b, 2017a, 2019a).

## Conclusion: Intersubjectivity as Right Brain-to-Right Brain Communications: Update and Clinical Applications

In the prior sections of this work, I described how the posterior right temporoparietal system is centrally involved in synchronized nonverbal communications. This adaptive function of the early forming core self is expressed in adulthood as a capacity to enter into intersubjective, reciprocal, right brain-to-right brain nonverbal interactions with another human, beneath the words. Recall previously cited research demonstrated “the importance of the right temporoparietal junction in *collaborative social interactions*” in a social context of a “*basic interpersonal interaction*.” In her recent research, Feldman et al. now assert that “brain coordination may be supported by the *non-verbal* rather than verbal aspects of social interactions” and that “brain-to-brain synchrony localizes to temporal-parietal regions, and highlight the role of attachment and social connectedness in the coordination of two brains” (Levy et al., 2017, p. 6, italics added). These descriptions clearly imply a shift from a one-person intrapsychic to a two-person interpersonal psychology, and a “two-person,” “two-brain” technology that can simultaneously measure two brains interacting in real time.

Toward that end, in the last decade, hyperscanning methodologies utilizing simultaneous electroencephalography (EEG), fMRI, magneto-encephalography (MEG), and near-infrared spectroscopy (NIRS) measurements have been created. In pioneering work, Montague et al. (2002) stated “studying social interactions by scanning the brain of just one person is analogous to studying synapses while observing either the presynaptic neuron or the postsynaptic neuron, but never both simultaneously” (p.1160). This technological advance now allows for a deeper understanding of a spontaneous social interaction between two dynamically synchronized, nonverbally communicating brains, including face-to-face, moment-to-moment right brain-to-right brain emotional communications at levels beneath awareness. The “ultrarapid” unconscious detection of a human face takes place in just 100 ms (Crouzet et al., 2010).

Inspired by the developmental research on sensitivity of social contingency in infants and mothers that used two synchronized video cameras (see earlier references), Dumas et al. (2010) offered a now classic dual EEG hyperscanning study of interbrain synchronization during a spontaneous social interaction between two adults, characterized by reciprocal nonverbal communication and turn-taking (a central feature of primary intersubjectivity). This methodology utilized a simultaneous measurement of brain activities of each member of a dyad during interpersonal communications, where “both participants are continuously active, each modifying their own actions in response to the continuously changing actions of their partner” (Dumas et al., 2010, p. 1). In this relational context, both share attention and compare cues of self and other’s actions. These researchers documented specific *changes in both brains* during nonverbal imitation, a central foundation of socialization and communication.

**Figure 2:**
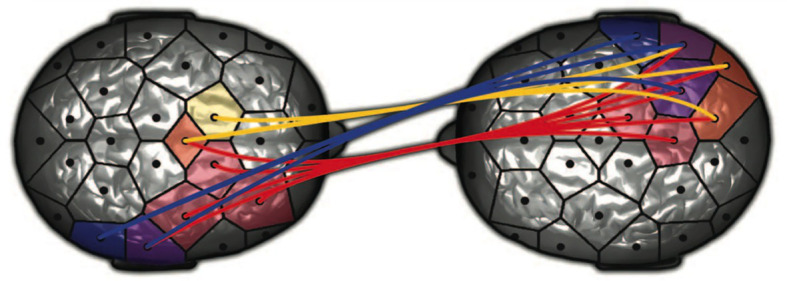
Top-down view of a right-lateralized interbrain synchronization of a co-created spontaneous nonverbal communication system. From “Toward a Two-Body Neuroscience” by Dumas (2011). Permission by Dumas.

Dumas et al. described the interbrain synchronization, on a milliseconds time scale, of the right centroparietal regions, a neuromarker of social coordination in both interacting partners (Tognoli et al., 2007) and synchronization between the right temporoparietal cortex of one partner and the right temporoparietal cortex of the other. Citing Decety and Lamm (2007), they pointed out that the right temporoparietal junction is known to be activated in social interactions, attention orientation, self-other discrimination, and the sense of agency. The top-down view of Figure 2 shows this interbrain synchronization lateralized to the right hemisphere of each member of a communicating dyad. Note that the figure depicts specifically right-lateralized interbrain synchronization, and what I have called a reciprocal right brain-to-right brain nonverbal communication system. This dynamic synchronized right TPJ-to-right TPJ “basic interpersonal interaction” operates “outside of conscious awareness (Chelazzi et al., 2018) as a “two-person unconscious” (Lyons-Ruth, 1999), a relational unconscious nonverbally communicating with another relational unconscious. In turn, this intersubjective interaction between the mind of one subjective self and another subjective self co-creates an emotionally-energized intersubjective field between them (Schore, 1994/2016, 2003a,b, 2012a, 2019a,b).

For three decades, my work on right brain-to-right brain nonverbal communication has described the critical role of intersubjectivity in the co-created mother-infant and therapist-patient relationship (Schore, 1994/2016, 2003b, 2012a, 2019a). Recall the description of Trevarthen (1993) of protoconversation occurring between “minds in expressive bodies” (p. 126). There is a long tradition in psychoanalytic psychotherapy that conceptualizes intersubjectivity as an unconscious interaction between the mind of the clinician and the mind of a patient (Benjamin, 2017; Yakeley, 2018). With an emphasis on the patient’s subjective experience, the focus is on changes in emotional and relational functions expressed in the therapeutic relationship, the product of the interaction of the patient’s mind/body and the therapist’s mind/body. Due to the current intense interest of both researchers and clinicians in early development in the primacy of affect, and in “implicit” (unconscious) phenomena that operate below levels of awareness, not only psychodynamic but also all forms of treatment now stress the role of rapid emotional communications between the minds of both members of the therapeutic alliance. This intersubjective context of nonverbal communication allows the patient and the empathic clinician to “make sense of another’s mind.”

Like the developmental process of attachment, intersubjectivity is now seen as a critical construct within psychotherapy. Although Trevarthen stressed the role of intersubjectivity in positively charged play states, psychotherapists also address the nonverbal intersubjective communications of negatively valenced and even traumatic emotional states (Schore, 2009, 2013). Indeed, early stressful ruptures of the attachment bond that are routinely not followed by relational repair are commonly found in patients with an early history of right brain attachment stressors. In my ongoing work, I continue to offer clinical and interdisciplinary research evidence demonstrating that all psychiatric disorders show deficits in right brain affect regulation, and that the right hemisphere is dominant in psychotherapy (Schore, 1994/2016, 2003b, 2012a, 2014a, 2019a).

There is now general acceptance that intersubjective relational deficits as well as affect dysregulation are a central focus of all forms of infant, child, adolescent, adult, and group psychotherapy (see Schore, 1997, 2000, 2002, 2003b, 2019b, 2020). In terms of the dyadic psychotherapeutic relationship, right brain deficits of intersubjectivity are expressed in an inability, especially under relational stress, to nonverbally emotionally communicate and interpersonally synchronize with another brain (e.g., see Schore, 2014b for deficits of intersubjectivity in autism spectrum and severe attachment disorders). It has long been established that stress is defined as an asynchrony in an interactional sequence, and thus “a period of synchrony, following the period of stress, provides a ‘recovery’ period” (Chapple, 1970, p. 631). Synchronized rupture and repair of the emotional attachment bond between the patient and therapist is thus an essential interpersonal neurobiological mechanism of the treatment (Schore, 1994/2016, 2003b, 2012a, 2019b). In addition to the patient’s symptomatology, these intersubjective difficulties lie at the core of the treatment of the patient’s relational deficits that operate outside of conscious awareness.

Toward that end, I continue to offer current neuroscience data and clinical material to describe how the co-created psychotherapeutic relationship acts as an intersubjective social context of spontaneous reciprocal nonverbal emotional communications of face, prosody, and gestures within the emerging therapeutic alliance (Schore, 2012a, 2019a). These dynamically synchronized moment-to-moment, right brain-to-right brain communications of emotional self-states between two minds take place in the present moment, a time frame of fractions of a second to 2–3 s. In recent writings on physiological synchrony in psychotherapy, Tschacher and Meier (2019) assert “Synchrony is generally defined as the social coupling of two (or more) individuals in the here-and-now of a communication context that emerges *alongside, and in addition to, their verbal exchanges*” (p. 558, italics added). This right-lateralized *nonverbal* psychobiological system, operating beneath levels of conscious awareness, intersubjectively synchronizes with another “emotional” right brain *that is tuned to receive these right brain-to-right brain communications*, clearly implying that, when working with the patient’s dysregulated emotions, the empathic clinician needs to transiently shift dominance from the explicit left brain to the implicit right brain. In recent research on “right hemispheric dominance in nonconscious processing,” Chen and Hsiao (2014) demonstrate that the left hemisphere plays a greater role in processing explicit knowledge, whereas the right hemisphere has an advantage in shaping behavior with implicit information.

In “heightened affective moments” of a psychotherapy session, the intuitive therapist implicitly “surrenders” into a callosal shift from the left hemispheric posterior temporoparietal system in Wernicke’s receptive language area that processes grammatical processing, semantic knowledge, and syntax in verbal conversations, to the right hemispheric posterior temporoparietal system that intersubjectively processes nonverbal emotional protoconversations. The key clinical ability of the empathic psychobiologically attuned therapist is not to intellectually understand the patient but to emotionally listen to and subjectively feel the patient. This transient reversal of hemispheric dominance allows the “open and receptive” clinician, in an implicit state of “therapeutic presence” and “wide-ranging,” “evenly-suspended,” and “free-floating attention” to listen beneath the patient’s words (see Schore, 2019b). This therapeutic intersubjective context allows the empathic therapist to emotionally *recognize* the patient and enables the patient’s right brain subjective self to emotionally experience *feeling felt* by the therapist (Schore, 1994/2016, 2012a, 2019a). In this manner, the emotionally sensitive therapist, *from the first point of contact in the first session*, is implicitly learning the nonverbal moment-to-moment rhythmic structures of the patient’s internal states and is relatively flexibly and fluidly modifying her own behavior to synchronize with that structure, thereby co-creating an interpersonal context for the organization of the right brain-to-right brain therapeutic alliance.

In the clinical literature, Meares (2012) directly refers to Trevarthen’s intersubjective protoconversation and asserts that the dynamic “interplay between two right brains provides a structure for the therapeutic relationship” (p. 312). Face-to-face interpersonal synchronization of right brain patterns enables the therapeutic dyad to intersubjectively communicate and implicitly share their conscious *and* unconscious emotional states in what I have termed “a conversation between limbic systems,” a “spontaneous emotion-laden conversation” (Schore, 2012a). This right-lateralized interpersonal neurobiological mechanism allows the clinician to act as an interactive regulator of the patient’s emotional states, which in turn facilitates a reduction in the patient’s presenting symptomatology. In this work, the clinician interactively downregulates negative effect in stress-reducing therapeutic contexts and upregulates positive effect in playful therapeutic contexts.

From the perspective of modern attachment theory (Schore and Schore, 2008) patients’ organized and disorganized attachment styles refer not only to different implicit strategies of emotion regulation, but also to different abilities for entering into intersubjective synchronized right-lateralized nonverbal emotional communications with others. These essential right brain functions are expressed in the therapeutic relationship, and thereby available for change. Indeed, the therapeutic working relationship co-created by the patient and therapist has long been known to be a major vector of psychotherapeutic change processes. In a recent special issue of the journal *Psychotherapy* entitled “Evidence-based psychotherapy relationships,” the editors Norcross and Lambert (2018) assert, “decades of research evidence and clinical experience converge: the psychotherapy *relationship* makes substantial and consistent contributions to outcome independent of the treatment” (p. 313, italics added). More than mastering techniques the skilled therapist is an expert in working directly with right brain emotions and interpersonal relationships. For clinical applications of regulation theory to working in the co-constructed relationship between the patient and therapist, especially within stressful rupture and repair interactions, I refer the reader to my latest clinical volume, *Right Brain Psychotherapy* (Schore, 2019b).

Very recent technological advances in neuroscience now allow for a deeper understanding of the interactions of two right brain systems in the therapeutic *relationship* or working alliance. Psychotherapy researchers are also now using functional near-infrared spectroscopy (fNIRS) hyperscanning, a non-invasive, safe, portable, low-cost imaging modality in order to study the brains of both patients and therapists during face-to-face psychotherapy in the research laboratory (Zhang et al., 2018). Citing previous studies showing interpersonal synchrony in the right temporoparietal junction during a collaborative face-to-face interaction (Tang et al., 2016), and in a context of social connectedness (Kinreich et al., 2017), their methodology also included subjective measures of the “bond or positive personal attachments” within the working alliance, that represent “the connection between client and counselors.”

During the 40-min session the therapeutic dyad discussed moderately stressful “developmental issues” and “interpersonal relationships” and focused on “the client’s emotional states or personal troubles.” These investigators document better working alliances and increased interbrain synchronization in specifically the right temporo-parietal junction between clients and counselors during the *first session* of *psychological counseling*. This effect was not found when they were just chatting. They conclude that this work supports an interpersonal synchrony model of psychotherapy, which dictates “the more tightly the client and counselor’s brains are coupled, the better the alliance” (Koole and Tschacher, 2016, p. 7). The authors further suggest that this interbrain synchronization of the temporoparietal system in the patient’s and therapists’ right brains plays an important role in building a positive relationship in the emerging therapeutic alliance. The authors point out that fNIRS is only able to detect changes in cortical blood flow, and thus it cannot probe subcortical structures, specifically mentioning the amygdala, insula, and anterior cingulate medial frontal cortex that are synaptically connected with the right temporoparietal cortex. That said fMRI studies during live face-to-face social interaction shows “greater activation in brain regions involved in social cognition and reward, including the right temporoparietal junction, anterior cingulate cortex, right superior temporal sulcus, ventral striatum, and amygdala” (Redcay et al., 2010, p. 1639).

In a more recent publication, this laboratory offers another fNIRS hyperscanning study of interactive brain synchronization during the interpersonal communication process within the first psychotherapeutic session, specifically exploring the role of the counselor’s experience in building a therapeutic alliance with the client (Zhang et al., 2020). The work addresses the widely accepted clinical principle that an effective therapeutic relationship or working alliance is the most common and essential therapeutic factor in the clinical and counseling literatures (Wampold and Imel, 2015), and that the establishment of an effective therapeutic relationship is an essential component of therapeutic expertise (Hill et al., 2017). Toward that end, experienced counselors with an “integrative orientation” based on more than one theoretical psychotherapeutic orientation and 600–4,000 h of clinical experience were compared with novice counselors in terms of a capacity for interpersonal brain synchronization.

These authors describe how in the face-to-face therapeutic context of the psychotherapy session reciprocal intersubjective communications represent an interpersonal context in which both members observed each other nonverbal cues, facial expressions, and gestures. Significant increases in interpersonal brain synchronization were especially evident when the clinician had more psychotherapy experience. They report that in the session the clinician’s ability to specifically focus on emotional states and to interpersonally synchronize with the client is an expression of therapeutic expertise, and that interpersonal brain synchronization is therefore an essential “*nonverbal skill* to help to improve the working alliance” (Zhang et al., 2020, p. 103, italics added). The authors also note that “experienced psychotherapists reported that they used moment-to-moment cues (e.g., emotional expression and body postures) and tried to be attentive to their clients reactions” and conclude that “expert/experienced counselors must be able to adapt to different types of clients, as well as being responsive and collaborative” (p. 8). Importantly, they again document that, during the session synchronous brain activity was seen in specifically the right temporoparietal junction of both members of the therapeutic dyad, *the exact same right-lateralized interpersonal brain synchronization and intersubjective right brain-to-right brain communication as the Dumas pattern in Figure 2*.

It should be noted that the intersubjective motivational system and the attachment motivational system are both central mechanisms involved in psychotherapeutic change, and that both are involved in adaptive right brain interpersonal neurobiological functions. Developmentally, the former system, located in the right temporoparietal regions and its subcortical connections enters a critical period of maturation at the beginning of the 1st year, while the latter, located in the right orbitofrontal regions and its more extensive cortical and subcortical connections, at the end of the 1st year (Schore, 1994/2016, 2003a,b, 2012a, 2019a,b). In the second year, the posterior right temporoparietal cortical areas form reciprocal bidirectional synaptic connections with the anterior right orbitofrontal cortical areas, the hierarchical apex of the limbic system and the locus of the attachment control system and the most complex affect and stress regulating mechanisms, thereby allowing the right brain to act as an integrative self-regulating system.

The psychobiologically simpler early maturing right-lateralized intersubjective system of reciprocal nonverbal *communication* between two right minds structurally and functionally evolves at the beginning the human brain growth spurt, in contrast to the more complex later maturing right-lateralized system of *regulation* of emotional states that structurally and functionally evolves at the end of the growth spurt. Throughout the life span the early developing capacity of intersubjectivity acts as a right-lateralized nonverbal *positive* emotion *communicating* and *mutually regulating system*, while the later maturing attachment system builds upon the intersubjective system and functions as an adaptive right brain regulation system that can *self-regulate and interactively regulate* positive *and* negative emotions. Thus, I have described how interactively regulated affect transactions that maximize positive and minimize negative affect cocreate a secure attachment bond between mother and infant. The construct of interpersonal synchrony is a central communicational element of both right brain intersubjective protoconversation and attachment dynamics. The evolutionary mechanism of attachment fundamentally represents the regulation of biological synchronicity between *and* within organisms. These dual right brain processes underly the right-lateralized subjective self’s capacity for *communicating* with other minds, intersubjectivity, as well as for attachment, interactively *regulating* emotion between and within brains, minds, and bodies.

## Data Availability Statement

The original contributions presented in the study are included in the article/supplementary material, and further inquiries can be directed to the corresponding author.

## Author Contributions

The author confirms being the sole contributor of this work and has approved it for publication.

### Conflict of Interest

The author declares that the research was conducted in the absence of any commercial or financial relationships that could be construed as a potential conflict of interest.
